# Overlapping

**Published:** 1892-03-05

**Authors:** 


### The Hospital

A Weekly Journal of

Science, Medicine, Nursing, and Philanthropy.

*For Hospitals, Asylums, Infirmaries, Dispensaries, Medical Practitioners, Students, Nurses, and the Charitable Public.*

Vol. XI.—No. 284.] [March 5, 1892.

### OVERLAPPING.

The Archbishop of Canterbury has found out that there is a good deal of overlapping in certain forms of charity. No experienced philanthropist will be surprised to hear of the Archbishop's discovery. His Grace presided at a Conference of those interested in the work of the Children's Country Holidays Fund at Lambeth Palace last Saturday. He stated that it had been proved beyond contradiction, last summer, that there were "plenty of people who subsisted entirely upon the rival kindness of different religious denominations—people who did no work, and who would not work because they could most thoroughly depend on the different religious denominations sending their district visitors round and giving them quite enough to go on with until they professed another denomination to the next visitor."

The readiness to "profess another denomination" is a veiy familiar fact to church visitors, and reveals an aspect of English character which is by no means encouraging. But, after all, there are, even among the very poor, a good many men and women who preserve the sturdy independence of their birthright; and it is eminently desirable, in discussing such a question as the overlapping of charity, that one should see only the actual facts as they are, and carefully avoid all sorts ef exaggeration. The late Dr. Raleigh once told a story at a public meeting which well illustrates the more independent attitude of some of the industrious poor. A Sunday morning visitor at the East-end of London knocked at the door of a small house, and was answered somewhat more promptly than he anticipated by a determined-looking person, who greeted him with an interrogatory and very forbidding "Well?" The visitor was a little taken aback; but, summoning courage, he said, in a very conciliatory tone, "Will you take a tract, my good woman?" "I'm not a good woman, and I won't take a track. I have had five religions at me this morning." Dr. Raleigh expressed much sympathy with that British matron, and most of us will cordially agree with him. There is no doubt that a majority, perhaps a very large majority, of even the less prosperous members of the working classes, would very gladly fight their own way in the world, and dispense with charitable aid altogether. But it is equally true that large numbers, even of the most industrious and independent, do often need the practical help of charity, and would, but for that help, experience much distress which they are now happily saved from, and often be defeated in life's struggle long before the period of old age. Charity has a noble work to do, and if she sometimes works with closed eyes when she ought to open them wide with suspicion, she is not the only blind activity in this complex and half-regulated world.

A timid person might well shrink from dealing with the overlapping of charitable organisation and effort. But timidity in a thickly-peopled and variously-organised State like our own is not the most commendable of the virtues. It is quite imperative that somebody should make an effort to improve the existing state of things. The Children's Country Holidays Fund is but one of many institutions in which good work is done twice or thrice over which ought only to be done once. Certain hospitals, which command an aggregate income many times greater than that of the Country Holidays Fund, are notorious offenders in the way of overlapping. Whilst every hospital fights for its own hand, as if there were no other similar institution in existence, this evil is bound to continue.

A few days ago the Members and Fellows of the Institute of Actuaries met to discuss a paper which was entitled, "An attempt to measure the extra risk arising from a consumptive family history when the life proposed for assurance is physically sound and healthy." There was a general consensus of opinion among the actuaries and medical men present that the real difficulty consisted, not in the measurement of the extra risk, but in persuading the proposer to admit the correctness of the measurement. When the doctor and the actuary of a given life office decide that a particular proposer ought to pay a fifteen or twenty years extra because his father or his mother happens to have died of consumption, the proposer when he is informed of the decision will not unfrequently demur, and sometimes with considerable indignation. Instead of accepting with grateful docility the terms offered to him by the assuring office, he marches off with outrageous unreasonableness to the competing institution on the opposite side of the street. It was quite pathetic to notice how the insurance officials present, more especially the actuaries, bewailed this degenerate development of the times. "If the different offices could only combine," sighed one. "If we only had the wisdom to work together," echoed another. "If some of the competing offices would only desist from the degrading sinuosities of their competing methods," reiterated a third, all would be well; the offices would be amply protected and bad lives would be promptly relegated to the cold and desolate shades where the non-insurable population have their abode. There were some present who felt that the abolition of competition might be all very well from the point of view of the insurance offices, but by no means so well from the standpoint of the insuring public.

There is a parallel to be drawn between these competing life offices and the metropolitan hospitals which overlap and strive to outdo one another in all directions. But there is this difference between the two sets of institutions. The competition among the various insurance offices may be bad for the offices and their shareholders, but it is undoubtedly good for the insuring public. It tends to lower rates, to increase facilities of every kind, and thus to promote thrift on all hands. But the competition among hospitals and other charities is injurious both to the charities and the public alike. The competing methods employed are very costly; the overlapping is still more wasteful, and the unfortunate public has to supply the resources for both overlapping and strained competition. So far as the insurance offices are concerned, we may be glad that they cannot combine; but in the very different case of hospitals and charities, we insist that they ought to combine. Nay, if they will not combine of themselves, it is probable that in no long time they will be compulsorily combined from without.

An outsider of candid mind might not unreasonably ask how it is that the hospitals of the metropolis are so determined to fight every one for its own hand, as if, in the picturesque language of the Old Testament, "it lived alone in the midst of the earth"? There is only one answer to this question. It is "human nature" that is at fault; unregulated human nature; the primitive instincts asserting themselves. Why should one hospital take thought for another? Why should one charity play second fiddle to another? Why should the "Royal Hospital for Diseases of the Pinna of the Ear," with two beds and a children's cot, sacrifice its independence to the "bloated magnificence" of a St. Thomas's or a Guy's? This is where the hopelessness of the business comes in. When you have a dozen microscopic hospitals adumbrating after this defiant fashion you feel that the hospital world, like the great world of which it is a microcosm, is in some aspects a comedy rather than a tragedy.

The *Times* the other day, lamenting the riots at Berlin, and the spirit of carping discontent prevalent throughout the German Empire, expressed the conviction that if some external danger were to threaten the safety of the Fatherland, discontents and disagreements would promptly disappear and ranks would be joined up in patriotic defiance in a moment. No doubt that would be so. The same might probably be said with truth of the jealousies and contentions which divide the hospital world asunder. Those who stand in the front ranks of discontent and opposition would lay down the weapons with which they have assailed their comrades, and don the nobler armour of a just and reasonable defence.

But if hospital managers and officials were very far-seeing people they would not wait for the combining energy of a hostile attack from the outside. It is not a foolish, but a wise thing to "set one's house in order." It is not a mark of high, but of low development to live in the midst of chaos and to be content with it. We hesitate to put the saddle of unreasonable dissidence on any particular horse. But we cannot forbear to express the conviction that if the honorary managers of hospitals, as well as the physicians and surgeons, would unite, as the secretaries have recently shown a disposition to do, a brighter day would dawn. If all charitable persons and institutions, indeed, had but a little more of Mr. Matthew Arnold's "sweet reasonableness," a great many of the difficulties of hospitals would vanish immediately.

